# Interstitial Lung Disease in Connective Tissue Diseases: Survival Patterns in a Population-Based Cohort

**DOI:** 10.3390/jcm10214830

**Published:** 2021-10-21

**Authors:** Charlotte Hyldgaard, Elisabeth Bendstrup, Alma Becic Pedersen, Lars Pedersen, Torkell Ellingsen

**Affiliations:** 1 Diagnostic Centre, Silkeborg Regional Hospital, University Clinic for Innovative Patient Pathways, Aarhus University, 8600 Silkeborg, Denmark; torkell.ellingsen@rsyd.dk; 2 Centre for Rare Lung Diseases, Department of Respiratory Diseases and Allergy, Aarhus University Hospital, 8200 Aarhus, Denmark; karbends@rm.dk; 3 Department of Clinical Epidemiology, Aarhus University Hospital, 8200 Aarhus, Denmark; abp@clin.au.dk (A.B.P.); lap@clin.au.dk (L.P.); 4 Rheumatology Research Unit, Odense University Hospital, University of Southern Denmark, 5000 Odense, Denmark

**Keywords:** interstitial lung disease, connective tissue disease, pulmonary hypertension, clinical epidemiology, rare lung disease

## Abstract

Objectives: Interstitial lung disease (ILD) is associated with impaired survival among patients with connective tissue diseases (CTDs), but population-based data on the frequency of ILD and pulmonary hypertension (PH) in different CTD subtypes and the impact on survival are sparse. Methods: We included patients with a first-time ICD-10 diagnosis of systemic sclerosis (SSc), mixed connective tissue disease (MCTD), myositis, systemic lupus erythematosus (SLE), or Sjögren’s disease registered in the Danish National Patient Registry between 2000 and 2015. Among these, we identified patients with ILD and PH. Using Kaplan–Meier analysis, we assessed survival for the five subtypes of CTD ± ILD and compared survival among CTD patients overall ± ILD with survival in the general population ± ILD. Results: We identified 11,731 patients with a diagnosis of CTD; 637 (5.4%) had a diagnosis of ILD. The proportion of patients with ILD was higher in SSc (13.4%) and MCTD (9.1%) than in myositis (6.0%), SLE (4.1%), and Sjögren (2.8%). Fifty-one percent were diagnosed with ILD in their fifties and sixties. PH was more frequent in SSc (7.5%) and MCTD (4.1%). Five-year survival was 73.3% (66.7–80.6) in SSc-ILD, 81.0% (69.0–95.1) in MCTD-ILD, 84.7% (77.3–92.9) in myositis-ILD, 83.5% (76.2–91.5) in SLE-ILD, and 84.7 (78.4–91.6) in Sjögren-associated ILD. Survival in CTD-ILD overall was impaired for all age groups compared with CTD alone. Age-stratified survival was comparable between CTD-ILD and ILD in the general population. The survival gap between ILD and non-ILD increased with age. Conclusion: Survival was comparable between different CTD-ILD subtypes and comparable to survival in non-CTD-ILD.

## 1. Introduction

Interstitial lung disease (ILD) is a serious manifestation of connective tissue diseases (CTD) associated with increased morbidity and mortality [1]. The clinical spectrum ranges from mild, self-limiting disease to progressive irreversible pulmonary fibrosis.

Population-level data on CTD-ILD are sparse and reflect the population studied, referral patterns, and whether the focus is clinically manifest ILD or subclinical disease with high-resolution computed tomography (HRCT) findings alone. Recent studies in systemic sclerosis (SSc) reported that 24–36% of the patients develop ILD [2,3,4]. A Norwegian study reported ILD on HRCT in 50% of newly diagnosed patients, and 11.8% had fibrosis that exceeded 10% of the total lung volume on HRCT [5]. In a recent report from the EUSTAR SSc database, 38% had signs of ILD [6].

In mixed connective tissue disease (MCTD), a frequency of ILD between 27 and 53% was reported after 10–12.5 years of follow-up [7,8,9], and in myositis, the frequency of ILD was 11–20% in large cohorts from referral centers [10,11,12].

In systemic lupus erythematosus (SLE), clinically apparent ILD is less common with reported frequencies ranging from 2.5% in a population-based study to 11% in data from a referral centre [13,14]. A study from Denmark reported that 4% of a multicentre SLE-cohort developed ILD within one year from diagnosis and 8% did during follow-up [15]. In Sjögren’s disease, the frequency of ILD was 11% in a multicentre study from Spain [16] and 27% in a population-based study from Norway [17].

Some patients with CTD-ILD develop progressive fibrotic disease with serious lung function impairment and reduced survival. The proportion of patients with CTD-ILD with a progressive phenotype has not been established; these patients may benefit from antifibrotic treatment [18,19].

Pulmonary hypertension (PH) is also associated with impaired survival in CTD. In SSc, pulmonary arterial hypertension (WHO group I) was reported in 24.1% and PH coexisting with ILD (WHO group III) PH in 55.2% [20]. Studies of the frequency of PH in CTD, especially non-SSc CTD, are sparse.

The aim of the present study was (1) to provide an overview of CTD, CTD-associated ILD, and PH in a population-based cohort using high-quality health registry data; (2) to assess age-related survival in patients with CTD and CTD-ILD compared with the general population, and (3) to assess survival in the five subtypes of CTD with and without ILD.

## 2. Methods

### 2.1. Setting and Data Sources

The study was conducted in Denmark in a population of 5.6 million persons at risk (2016) using prospectively collected data from population-based databases.

The data set was obtained by linking data from the Danish Civil Registration System (CRS) [21] and the Danish National Patient Registry (DNPR) which contains contacts and diagnoses from all hospitalisations and outpatient contacts [22].

### 2.2. Study Population and Study Period

We used the DNPR to identify all patients with a first-time diagnosis of CTD between 1 January 2000 and 31 December 2015. We used the ICD-10 group codes M34.0–M34.9 Systemic sclerosis, M35.1 Mixed connective tissue disease, M33.0–M33.9 Polymyositis/dermatomyositis, M32.0–M32.9 Systemic lupus erythematosus, and M35.0 Sjögren’s disease. We excluded all patients with a diagnosis of the relevant CTDs before 2000. We focused on the small CTDs without inclusion of rheumatoid arthritis, because of the differences in disease characteristics and management strategies.

Information on diagnoses of ILD and pulmonary hypertension (PH) was obtained from the DNPR using hospital contacts since 1994 in order to identify patients diagnosed with ILD prior to CTD as well as after CTD. The ICD-10 codes used were J84.0–J84.9 Other ILD and I27.0–I27.9 Pulmonary hypertension. ILD in the general population was identified using J84.0–J84.9. Information on death from all causes was obtained from the CRS. Patients were grouped according to the first CTD diagnosis.

The study was approved by The Danish Data Protection Agency (1-16-02-277-16) and the Danish Health Data Authority (FSEID-00002213).

### 2.3. Data Analysis

We computed the age and gender distribution for CTD and CTD-ILD overall and for each subgroup of CTD. Survival for CTD patients with and without ILD was assessed using Kaplan–Meier analysis with age as time origin and compared to the general population with and without ILD. All patients who were 40 years of age at any time during the study period were included in the age 40 assessment, etc.

We assessed survival for each subtype of CTD with time of CTD diagnosis as time origin. For CTD-ILD, we assessed survival for each subtype of CTD-ILD with time of ILD diagnosis as time origin.

## 3. Results

**Figure 1:**
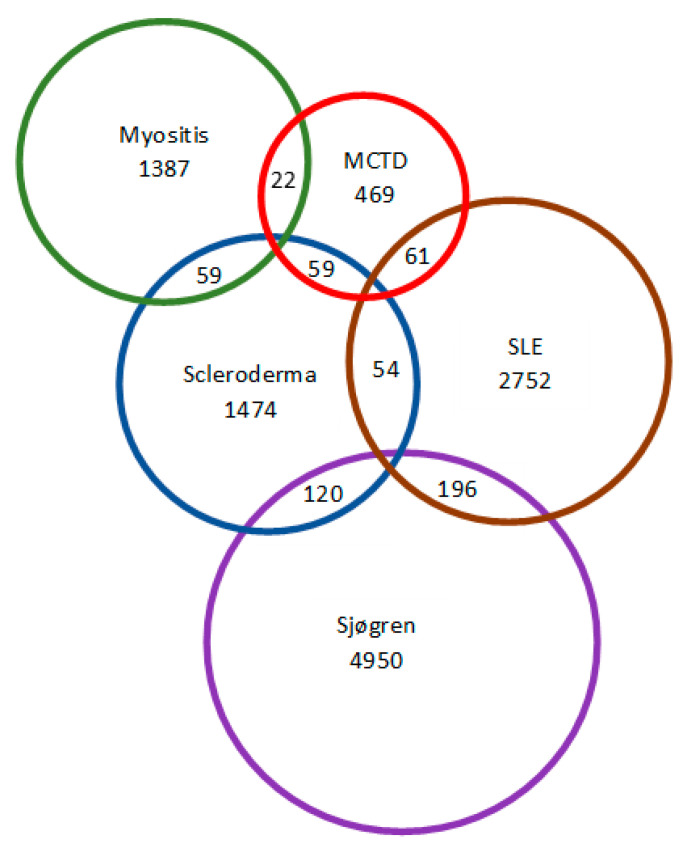
Diagram showing overlapping CTD diagnoses and number of patients with more than one CTD diagnosis during the study period.

Between 2000 and 2015, we identified 11,731 incident patients with a CTD diagnosis. Forty-two percent were in their fifties and sixties when diagnosed with CTD, and 79% of the patients were female. Patients with SLE and MCTD were younger at the time of diagnosis (mean age 47.2 and 47.6 years). Female predominance was seen for all CTD subtypes, although this was less prominent in myositis with 55% female patients (Table 1). The majority of patients with CTD (11,009/11,731, 93.8%) had only one CTD diagnosis during the study period; 5.6% (657/11,731) had two different CTD diagnoses, and 0.55% (62/11,731) had three or more different CTD diagnoses (Figure 1).

Combinations not shown are: Myositis + SLE *n* = 24, Myositis + Sjögren *n* = 24, MCTD + Sjögren *n* = 38, three diagnoses *n* = 62, and four diagnoses *n* = 3.

### 3.1. CTD-ILD and PH

Among all incident patients with CTD in the study period, a total of 637 patients (5.4%) had a diagnosis of ILD. The percentage of patients with ILD was higher in SSc (13.4%), MCTD (9.1%) and myositis (6.0%) compared with patients with SLE (4.1%) and Sjögren’s disease (2.8%). PH was also more frequent among patients with SSc (7.5%) than in the other CTDs. A total of 2.2% of all patients with CTD had a diagnosis of PH, but among patients with CTD-ILD, 14.8% developed PH. PH was especially frequent in SSc-ILD (24.1%) and MCTD-ILD (20.8%) (Table 2).

A diagnosis of PH was assigned mainly to patients in their sixties and seventies (149/263, 57%), although PH was seen earlier among patients with co-existing ILD; 54% of PH-ILD patients were in their fifties and sixties.

The mean age was 56.7 years for CTD-ILD overall, ranging from 53.9 years in SLE to 63.9 in Sjögren’s disease. During the study period, the mean age at CTD-ILD diagnosis gradually increased from 51.9 years in 2000–2003 to 59.4 years in 2013–2015 (Table 3).

Thirty-three percent (212/637) of the patients were diagnosed with ILD before CTD, and 56% were diagnosed with ILD within two years before or after the CTD diagnosis. The percentage of patients diagnosed with ILD in the first two years after the CTD diagnosis increased during the study period. Similarly, an increasing number of patients diagnosed with ILD before CTD, received the CTD diagnosis within two years after ILD (Supplementary Table S1).

### 3.2. Survival in CTD and CTD-ILD

**Figure 2:**
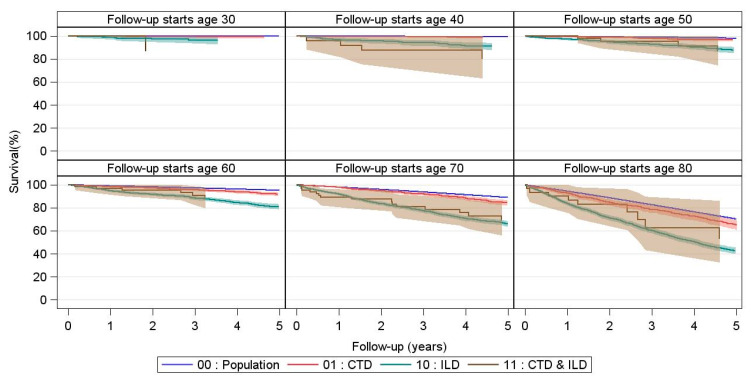
Survival curves for CTD patients with and without ILD stratified by age compared with non-CTD ILD patients and the general population in the same age stratum.

We compared survival in CTD, CTD-ILD, other ILDs not associated with CTD, and the general population in an age-stratified model (Figure 2). For all age groups, CTD-ILD was associated with impaired survival compared with CTD without ILD. Similarly, ILDs not associated with CTD were associated with impaired survival compared with the general population. Survival for CTD-ILD and other ILDs not associated with CTD was comparable for all age categories. The survival gap between individuals with ILD and individuals without ILD increased with increasing age, regardless of ILD being associated with CTD or not. An overview of the numbers at risk and the number of censored patients is shown in Supplementary Table S2.

**Figure 3:**
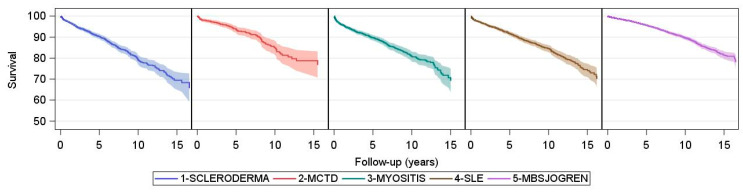
Survival in CTD. Kaplan–Meier survival curves for each CTD subtype with adjustment for age. Time origin is the date of CTD diagnosis. Shaded areas represent 95% confidence intervals.

The overall survival for the five CTD subgroups is shown in Figure 3.

All patients who were 40 years of age at any time during the study period were included in the age 40 assessment; all patients who were 50 years of age at any time during the study period were included in the age 50 assessment, and so on.

Comparison was made with (1) The general population without CTD or ILD and (2) Patients with ILD (and no CTD) in the general population. Shaded areas represent 95% confidence intervals.

**Figure 4:**
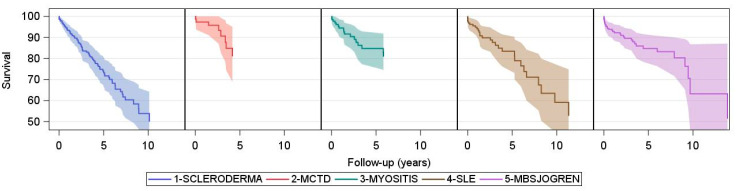
Survival in CTD-ILD. Kaplan–Meier survival curves for each CTD-ILD subtype with adjustment for age. Time origin is the date of ILD diagnosis. Shaded areas represent 95% confidence intervals.

For the five CTD-ILD subtypes, age-adjusted survival at five years was also comparable ranging from 73.3% (66.7–80.6) in SSc-ILD, 81.0% (69.0–95.1) in MCTD-ILD, 84.7% (77.3–92.9) in myositis-ILD, 83.5% (76.2–91.5) in SLE-ILD, to 84.7 (78.4–91.6) in Sjögren-associated ILD. SSc-ILD had the worst long-term outcome with adjusted ten-year survival of 53.8% (43.9–65.9). The estimates for the other subtypes are associated with greater statistical uncertainty (Figure 4).

## 4. Discussion

The study shows the distribution of ILD and PH for five different subtypes of CTDs based on a uniform approach allowing a comparison between the subtypes. The presence of ILD was associated with impaired survival for all five subtypes of CTD, but the impact on survival was comparable between the CTDs. Age-stratified survival was similar in CTD-ILD and other ILDs in the general population, and survival in ILD decreased with increasing age.

The percentage of patients with CTD-ILD was low compared to previous studies from referral centers and international disease registries [4,5,9,12], but the higher frequency of ILD in SSc and MCTD compared to other CTDs is in accordance with previous findings. Most studies of CTD-ILD include clinically significant ILD as well as subclinical disease, and no consensus exists on how to define clinically significant ILD. The health registry data used in the present study do not provide detailed information of clinical characteristics, but subclinical ILD is not likely to be included. However, the registry data reflect the burden of clinically significant ILD. The limitation is the lack of validation of the diagnoses at the individual level, but the uniform approach allows the comparison between different CTDs.

For all five subtypes of CTD, approximately one third of the patients were diagnosed with ILD before CTD, emphasising the importance of screening for symptoms and signs of CTD in patients with ILD, regardless of age. The time from CTD diagnosis to ILD diagnosis as well as the time from ILD to CTD diagnosis decreased during the study period. Increased awareness of ILD and improved access to HRCT scans and pulmonary function tests may have enabled earlier diagnosis, although the length of follow-up plays a role.

SSc-ILD was the largest group of CTD-ILD, and the highest rate of PH was also seen in SSc with 7.5% of the entire SSc cohort and 24% of the SSc-ILD patients. Long-term survival was lower in SSc-ILD than in the other CTD-ILDs, but short-term survival was comparable among the five CTD-ILD subgroups.

The mean age among patients with SSc-ILD in our study was 58.4 years and the average time between the SSc diagnosis and the ILD diagnosis was 2.7 years. Participants in previous large, randomised trials in SSc-ILD, SENSCIS and the Scleroderma Lung Study II, were 52–54 years on average at the time of enrollment, with a mean time from SSc diagnosis between 2.6 and 3.5 years, [23,24] and thus, slightly younger than the average patient in the present study.

The observed increase in the age at CTD-ILD diagnosis occurred gradually during the study period, but was only accompanied by a minor increase in the number of incident CTD-ILD diagnoses. This change cannot easily be explained, but it may reflect the general improvement in the management of the CTDs as well as concomitant comorbidities leading to an improved overall survival and an increasing prevalence of CTD.

In 38.4% of SSc-ILD patients, ILD occurred within the first two years after the SSc diagnosis, and in 32.5% of the patients, ILD occurred more than two years after the SSc diagnosis, contradicting the common assumption that ILD occurs within the first two years. However, the mean time between CTD and ILD diagnoses declined during the study period from 5.5 years in 2000–2003 to 1.5 years in 2013–2015, probably reflecting a trend towards systematic screening for ILD.

Our study showed a one-, three-, and five-year survival of 95%, 83%, and 73% and a median survival of nine years in SSc-ILD. In accordance with these findings, Morisset at al. reported a three-year survival of 72–86% in a cohort from two ILD centers [25]. In two other cohorts from SSc-ILD centers, Bouros et al. reported a five-year survival of 82–91% [26], and Guler et al. reported a five-year survival of approximately 80% and a median survival of 11.2 years [27]. In the study by Hoffmann-Vold et al., five year survival was 69% among patients who had ILD on HRCT at the time of SSc diagnosis, but FVC within the normal range [5]. Interestingly, the impact of ILD on survival is of a similar magnitude in the population-based Norwegian cohort as in the other SSc-ILD cohorts and in our health registry cohort.

The present study provides an overview of PH among patients with CTD. As expected, PH was relatively frequent in SSc with 7.5% of the patients diagnosed with PH during the follow–up period. In MCTD, 4.1% of the patients had PH, and in myositis, in SLE, and Sjögren’s disease, only 1.0–1.4% were diagnosed with PH. In a population-based study from the Netherlands, pulmonary arterial hypertension (PAH) or PH-ILD was present in 11% of individuals [28], and in a Norwegian cohort, PAH was diagnosed in 7% and ILD-PH in 4% of individuals based on right heart catheterization [29].

A study from Norway reported that 3.4% of patients with MCTD developed PH [30]. In SLE, a study from Spain reported PH in 2.4%, ILD in 2%, and acute lupus pneumonitis in 3.6% of the patients; these pulmonary manifestations had a major impact on survival [31]. These findings are in accordance with our study showing that ILD and PH are relatively rare in non-SSc CTD-ILD.

The overall pattern of survival in different age strata was the same for CTD-ILD and non CTD-ILD; significant survival impairment was observed in both groups. The survival gap between ILD and non-ILD increased with increasing age, which may be caused by the predominance of fibrotic ILD in older age, and thus a lower survival in this group. Unfortunately, questions of distribution between fibrotic and inflammatory ILD patterns, disease severity, and progression cannot be answered using registry data.

Our group has previously published a study of rheumatoid arthritis-associated ILD (RA-ILD) showing that RA-ILD was present in 2% of patients with RA based on diagnoses in the national patient registry [32]. The present study suggests that ILD is more frequent in other CTDs than in RA, especially in SSc and MCTD; however, survival was more severely impaired in RA-ILD than in CTD-ILD in the present study.

### Strengths and Limitations

The advantage of national registry data is the possibility of studying large cohorts with minimal selection bias and complete follow-up with respect to survival. We have chosen broad inclusion criteria for CTD and ILD based on the knowledge that the rare ILD and CTD diagnoses were assigned almost exclusively by pulmonologists and rheumatologists. This likely reduced the risk of misclassification compared to more common diseases, although an inherent risk persists. We used broad diagnostic codes for ILD based on our experience indicating that J848 (other ILD) and J849 (unspecified ILD) are often used for CTD-ILD. We also included J841 (ILD with fibrosis, including Idiopathic pulmonary fibrosis) to ensure the inclusion of patients misclassified with a diagnosis of idiopathic ILD.

The main limitation is the lack of case validation at an individual level. We did not have access to individual patient data such as pulmonary function, HRCT, or treatment, and validation studies of registry diagnoses of ILD in the registry do not exist. However, the validity of registry data for these rare diseases is supported by previous studies showing high positive predictive values of SSc and PH diagnoses in the DNPR [33,34]. Studies in a wide range of other diseases have validated data from the DNPR [35,36], which is extensively used in epidemiological research.

## 5. Conclusions

CTD-ILD was associated with a significant survival impairment compared with CTD without ILD, but the five-year mortality was comparable between CTD-ILD subtypes.

The present study shows the frequency of CTD-ILD at the population level, but registry data cannot distinguish between stable ILD, ILD responsive to anti-inflammatory therapies, or progressive ILD. Nevertheless, the data on incidence and survival for each of the CTD-ILD subtypes provide a useful estimate of the burden of clinically significant CTD-ILD.

Studies of disease behaviour are needed to clarify the frequency of progressive fibrotic CTD-ILD as well as risk factors for progression.

## Figures and Tables

**Table 1:** **CTD:** Age and gender distribution within each CTD subgroup and frequencies of each CTD diagnosis over time.

Variable	SSc		MCTD		Myositis		SLE		Mb Sjögren		Total
	N	%	N	%	N	%	N	%	N	%	N
Overall:	1766	100	582	100	1446	100	2867	100	5070	100	11,731
Mean age at CTD diagnosis, years (SD)	54.2 (17.4)		47.6 (21.3)		55.2 (17.7)		47.2 (18.0)		59.2 (17.4)		
Age groups											
00–19	85	4.8	66	11.3	94	6.5	189	6.6	40	0.8	474
20–29	96	5.4	72	12.4	73	5.0	372	13.0	179	3.5	792
30–39	169	9.6	84	14.4	132	9.1	499	17.4	377	7.4	1261
40–49	277	15.7	96	16.5	192	13.3	536	18.7	713	14.1	1814
50–59	430	24.3	76	13.1	277	19.2	515	18.0	1213	23.9	2511
60–69	390	22.1	84	14.4	328	22.7	425	14.8	1227	24.2	2454
70–79	248	14.0	62	10.7	255	17.6	241	8.4	931	18.4	1737
>80	71	4.0	42	7.3	95	6.6	90	3.1	390	7.7	688
Gender:											
Male	471	26.7	141	24.2	647	44.7	508	17.7	737	14.5	2504
Female	1295	73.3	441	75.8	799	55.3	2359	82.3	4333	85.5	9227
Year of diagnosis											
2000–2003	364	20.6	126	21.6	286	19.8	597	20.8	1024	20.2	2397
2004–2006	272	15.4	106	18.2	239	16.5	521	18.2	975	19.2	2113
2007–2009	338	19.1	101	17.4	248	17.2	544	19.0	830	16.4	2061
2010–2012	344	19.5	119	20.4	313	21.6	573	20.0	938	18.5	2287
2013–2015	448	25.4	130	22.3	360	24.9	632	22.0	1303	25.7	2873

CTD connective tissue disease, MCTD mixed connective tissue disease, SSc systemic sclerosis, SLE systemic lupus erythematosus.

**Table 2:** CTD-ILD: Cumulative frequencies of ILD, PH, and ILD with PH among patients with CTD.

	All CTD	Interstitial Lung Disease		Pulmonary Hypertension		ILD and PH		
	N	N	% of CTD Patients	N	% of CTD Patients	N	% of ILD Patients	% of All CTD Patients
SSc	1766	237	13.4	133	7.5	57	24.1	3.2
MCTD	582	53	9.1	24	4.1	11	20.8	1.9
Myositis	1446	87	6.0	20	1.4	5	5.7	0.3
SLE	2867	118	4.1	34	1.2	11	9.3	0.4
Mb Sjögren	5070	142	2.8	52	1.0	10	7.0	0.2
Total	11,731	637	5.4	263	2.2	94	14.8	0.8

CTD connective tissue disease, ILD interstitial lung disease, PH pulmonary hypertension, MCTD mixed connective tissue disease, SSc systemic sclerosis, SLE systemic lupus erythematosus.

**Table 3:** **CTD-ILD:** Age and gender distribution for each CTD-ILD subgroup.

	SSc- Associated ILD	MCTD-Associated ILD	Myositis-Associated ILD	SLE- Associated ILD	Mb Sjögren-Associated ILD
Total, N	237	53	87	118	142
Female gender, %	67.5	56.6	50.6	77.1	83.1
Mean age at CTD diagnosis, years (SD)	56.4 (13.3)	53.9 (17.8)	55.8 (16.7)	52.4 (18.0)	62.4 (13.2)
ILD before CTD, N (%)	69 (29)	18 (34)	34 (39)	33 (28)	58 (41)
ILD after CTD, N (%)	168 (71)	35 (66)	53 (61)	85 (72)	84 (59)
Mean age at ILD diagnosis, years (SD)	58.4 (12.8)	55.1 (16.8)	56.5 (16.4)	53.9 (18.0)	63.9 (12.5)
Mean age at ILD diagnosis if ILD before CTD, years (SD)	60.4 (12.2)	60.4 (14.9)	54.2 (15.6)	54.4 (16.2)	62.8 (13.3)
Mean age at ILD diagnosis if ILD after CTD, years (SD)	57.4 (13.0)	52.2 (17.3)	58.5 (17.0)	53.7 (19.0)	64.9 (11.8)
Mean time between CTD and ILD diagnosis, years (SD)	2.7 (3.6)	2.5 (3.0)	2.7 (3.7)	3.4 (4.2)	4.1 (4.1)

CTD connective tissue disease, ILD interstitial lung disease, SSc systemic sclerosis, MCTD mixed connective tissue disease, SLE systemic lupus erythematosus, SD standard deviation.

## Data Availability

Data not available.

## Supplementary Materials

The following are available online at https://www.mdpi.com/article/10.3390/jcm10214830/s1, Table S1: Time between CTD and ILD diagnoses, Table S2: Overview of the numbers at risk and the number of censored patients for the Kaplan-Meier survival analysis shown in Figure 2.
