# Supplementary material for: Interstitial Lung Disease in Connective Tissue Diseases: Survival Patterns in a Population-Based Cohort
Source: J Clin Med. 2021 Oct 21;10(21):4830. doi: 10.3390/jcm10214830 (PMC8584507; doi:10.3390/jcm10214830)
Supplement: Supplementary file 1 [file jcm-10-04830-s001.zip › jcm-1414169-supplementary.pdf]

Supplementary Table S1. Time between CTD and ILD diagnoses.

	Time since CTD								
	ILD more than Two Years before CTD		ILD within Two Years before CTD		ILD within Two Years after CTD		ILD more than Two Years after CTD		Total
	N	%	N	%	N	%	N	%	N
	63	9.9	149	23.4	208	32.7	217	34.1	637
**First CTD diagnosis**									
SSc	15	6.3	54	22.8	91	38.4	77	32.5	237
MCTD	4	7.5	14	26.4	20	37.7	15	28.3	53
Myositis	9	10.3	25	28.7	32	36.8	21	24.1	87
SLE	11	9.3	22	18.6	39	33.1	46	39.0	118
Mb Sjögren	24	16.9	34	23.9	26	18.3	58	40.8	142
**Year of CTD diagnosis**									
2000 - 2003	14	9.5	23	15.5	30	20.3	81	54.7	148
2004 - 2006	10	10.3	20	20.6	23	23.7	44	45.4	97
2007 - 2009	12	9.6	22	17.6	39	31.2	52	41.6	125
2010 - 2012	10	8.9	32	28.6	41	36.6	29	25.9	112
2013 - 2015	17	11.0	52	33.5	75	48.4	11	7.1	155

CTD connective tissue disease, ILD interstitial lung disease, MCTD mixed connective tissue disease, SSc systemic sclerosis, SLE systemic lupus erythematosus.

Supplementary Table S2. Overview of the numbers at risk and the number of censored patients for the Kaplan-Meier survival analysis shown in Figure 2.

	Group	Total	Events	Censored	Censored %	Survival % (95% CI)
**Age cohort 40**		879696	4784	874912	99.46	
	Population	878496	4741	873755	99.46	99.4 (99.3-99.4)
	ILD	400	32	368	92	90.6 (87.5-93.8)
	CTD	776	7	769	99.1	98.9 (98.1-99.7)
	CTD-ILD	24	4	20	83.33	80.0 (63.3-100.0)
**Age cohort 50**		850835	13684	837151	98.39	
	Population	848939	13576	835363	98.4	98.1 (98.0-98.1)
	ILD	749	75	674	89.99	87.6 (84.9-90.3)
	CTD	1102	29	1073	97.37	96.6 (95.3-97.8)
	CTD-ILD	45	4	41	91.11	86.5 (74.5-100.0)
**Age cohort 60**		795865	32188	763677	95.96	
	Population	792955	31862	761093	95.98	95.3 (95.2-95.3)
	ILD	1415	229	1186	83.82	80.7 (78.4-83.0)
	CTD	1431	91	1340	93.64	91.8 (90.2-93.5)
	CTD-ILD	64	6	58	90.63	88.2 (79.6-97.8)
**Age cohort 70**		586304	52618	533686	91.03	
	Population	582941	51897	531044	91.1	88.9 (88.9-89.0)
	ILD	1957	552	1405	71.79	65.9 (63.5-68.3)
	CTD	1342	154	1188	88.52	84.2 (81.8-86.6)
	CTD-ILD	64	15	49	76.56	68.8 (55.8-84.8)

**Age cohort 80**		311768	78341	233427	74.87	
	Population	309416	77359	232057	75	70.2 (70.0-70.4)
	ILD	1604	776	828	51.62	42.6 (39.9-45.5)
	CTD	718	197	521	72.56	64.8 (60.7-69.2)
	CTD-ILD	30	9	21	70	52.8 (32.3-86.1)
